# Overexpression of vesicle-associated membrane protein *PttVAP27-17* as a tool to improve biomass production and the overall saccharification yields in *Populus* trees

**DOI:** 10.1186/s13068-021-01895-0

**Published:** 2021-02-16

**Authors:** Madhavi Latha Gandla, Niklas Mähler, Sacha Escamez, Tomas Skotare, Ogonna Obudulu, Linus Möller, Ilka N. Abreu, Joakim Bygdell, Magnus Hertzberg, Torgeir R. Hvidsten, Thomas Moritz, Gunnar Wingsle, Johan Trygg, Hannele Tuominen, Leif J. Jönsson

**Affiliations:** 1 Department of Chemistry, Umeå University, 901 87, Umeå, Sweden; 2 Umeå Plant Science Centre, Department of Plant Physiology, Umeå University, 901 87 Umeå, Sweden; 3 Umeå Plant Science Centre, Department of Forest Genetics and Plant Physiology, Swedish University of Agricultural Sciences, 901 83 Umeå, Sweden; 4 SweTree Technologies, PO Box 7981, 907 19 Umeå, Sweden; 5 Present Address: Faculty of Chemistry, Biotechnology and Food Science, Norwegian University of Life Sciences, 1432 Ås, Norway; 6 Computational Life Science Cluster (CLiC), Department of Chemistry, Umeå University, Umeå, Sweden; 7 Present Address: Department of Microbiology and Immunology, Institute of Biomedicine, University of Gothenburg, 40530 Gothenburg, Sweden; 8 Present Address: Umeå Plant Science Centre, Department of Forest Genetics and Plant Physiology, Swedish University of Agricultural Sciences, 901 83 Umeå, Sweden

**Keywords:** *Populus*, Vesicle-associated membrane protein, VAMP, VAMP-associated protein, VAP27, Growth, Bioprocessing, Transcriptomics, Proteomics, Metabolomics

## Abstract

**Background:**

Bioconversion of wood into bioproducts and biofuels is hindered by the recalcitrance of woody raw material to bioprocesses such as enzymatic saccharification. Targeted modification of the chemical composition of the feedstock can improve saccharification but this gain is often abrogated by concomitant reduction in tree growth.

**Results:**

In this study, we report on transgenic hybrid aspen (*Populus tremula* × *tremuloides*) lines that showed potential to increase biomass production both in the greenhouse and after 5 years of growth in the field. The transgenic lines carried an overexpression construct for *Populus tremula* × *tremuloides* vesicle-associated membrane protein (VAMP)-associated protein *PttVAP27-17* that was selected from a gene-mining program for novel regulators of wood formation. Analytical-scale enzymatic saccharification without any pretreatment revealed for all greenhouse-grown transgenic lines, compared to the wild type, a 20–44% increase in the glucose yield per dry weight after enzymatic saccharification, even though it was statistically significant only for one line. The glucose yield after enzymatic saccharification with a prior hydrothermal pretreatment step with sulfuric acid was not increased in the greenhouse-grown transgenic trees on a dry-weight basis, but increased by 26–50% when calculated on a whole biomass basis in comparison to the wild-type control. Tendencies to increased glucose yields by up to 24% were present on a whole tree biomass basis after acidic pretreatment and enzymatic saccharification also in the transgenic trees grown for 5 years on the field when compared to the wild-type control.

**Conclusions:**

The results demonstrate the usefulness of gene-mining programs to identify novel genes with the potential to improve biofuel production in tree biotechnology programs. Furthermore, multi-omic analyses, including transcriptomic, proteomic and metabolomic analyses, performed here provide a toolbox for future studies on the function of VAP27 proteins in plants.

## Background

Wood and other lignocellulosic feedstocks can serve as an abundant renewable source of sugars and other platform molecules for the production of advanced biofuels, green chemicals, and bio-based materials. One way to deconstruct the biomass is through pretreatment and enzymatic saccharification, which results in the release of sugars from cellulose and hemicelluloses [1, 2]. In this process, the major challenge is the recalcitrance of the biomass mainly due to prevention of access of enzymes to cellulose by hemicelluloses and lignin, high degree of cellulose polymerization, covalent cross-linkages between lignin and hemicelluloses, and other factors that remain to be determined [1, 3, 4]. Although wood is rather well characterized chemically and anatomically, the knowledge on genetic pathways that could be manipulated to reduce feedstock recalcitrance without compromising the properties and formation of wood is still scarce [5–7]. Identification of novel genes and/or pathways that control wood properties and growth of trees, therefore, provides a foundation not only for genetic improvement of the quality and quantity of wood, but also for improved saccharification efficiency and convertibility of woody biomass.

Numerous initiatives have been taken to improve saccharification in forest feedstocks by transgenic means. The most commonly studied species belong to the *Populus* family, including poplar and aspen trees. Advantages of *Populus* trees include their high biomass production rate (25 Mg ha^−1^ year^−1^), genetic diversity, market opportunities, and advanced genomic tools [8–11]. A critical aspect of improving saccharification of *Populus* trees has been the risk for growth penalties when modifying wood properties for reduced recalcitrance. Even though improved saccharification of transgenic trees has been obtained without growth penalties [12–15], most often increased saccharification is associated with impaired growth [16–22]. It is, therefore, important to identify novel ways to reduce feedstock recalcitrance to saccharification by enzymatic hydrolysis. Furthermore, most of the above-mentioned examples of improved saccharification without growth penalties require re-examination under field conditions.

A large-scale gene-mining program was launched in hybrid aspen (*Populus tremula* × *tremuloides*) to identify genes improving wood properties and/or chemistry by transgenic means [23]. We screened a selected set of transgenic lines derived from this program to identify novel approaches to increase saccharification yields without concomitant growth penalties [22]. One of the most promising lines concerned modification of a *Populus VAP27-17* gene (Line BI-36, [22]). VAPs [vesicle-associated membrane protein (VAMP)-associated proteins] are well-conserved, eukaryotic proteins, which work as tethers at endoplasmic reticulum–plasma membrane contact sites (EPCSs) or between ER and endosomes [24, 25]. In the present study, the function of the *Populus tremula* × *tremuloides* VAP27-17 (PttVAP27-17) was characterized in transgenic hybrid aspen lines both in the greenhouse and in a 5-year field trial. Analyses of transgenic trees both in the greenhouse and for 5 years in the field showed that constitutive overexpression of *PttVAP27-17* has potential to increase the whole-tree saccharification yields due to increases in biomass production, making this approach interesting from a biotechnological point of view.

## Results

### A VAP27 gene family member is a candidate gene for improved saccharification

**Fig. 1:**
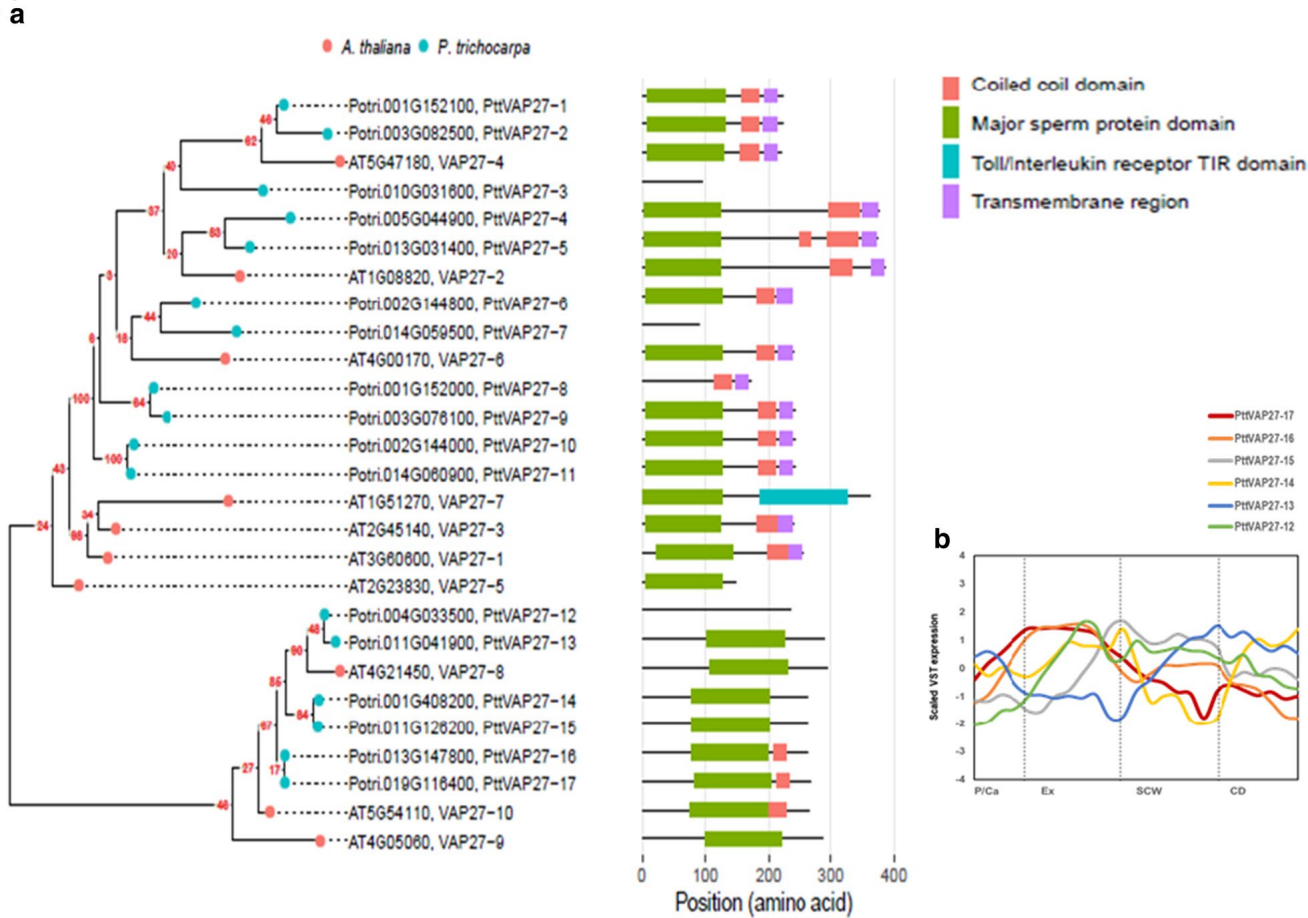
Phylogeny and expression profile of VAP27 proteins. **a** Phylogenetic analysis and protein domain characterization of VAP27 proteins in *A. thaliana* and *P. trichocarpa*. **b** The expression profile of the *PttVAP*27-*12, -13, -14, -15, -16* and* -17* genes in the *Populus* stem. Relative expression values throughout the different stages of wood development were obtained from the RNA sequencing data in the AspWood database [10]. Scaled VST expression indicates scaled variance-stabilizing transformation. *P/Ca* phloem/cambium, *Ex* expanding xylem, *SCW* maturing xylem, *CD* xylem cell death

In the course of our efforts to identify novel genes that improve bioprocessing properties of woody plants, we identified earlier a *Populus tremula* × *tremuloides* gene that encodes a member of the VAP27 gene family [22]. We first characterized the VAP27 gene families in *Populus trichocarpa* and *Arabidopsis thaliana* (*Arabidopsis* from here on) genomes. *Arabidopsis* genome contains 10 *VAP27* genes, which have been reported to cluster into three subfamilies: I, II, and III [25]. These genes were found to belong to two PLAZA (v4.0, [26]) gene families: HOM04D000560 and HOM04D001450. Seventeen *P. trichocarpa* genes (*PtrVAP27*) were identified in these two PLAZA gene families. Phylogenetic analysis of the corresponding *Arabidopsis* and *P. trichocarpa* proteins revealed a bigger group of proteins that included both the *Arabidopsis* subfamily I and III proteins and 11 homologous *P. trichocarpa* proteins (PttVAP27-1–PttVAP27-11), and a quite distantly related, smaller group containing the *Arabidopsis* subfamily II proteins together with six *P. trichocarpa* proteins (PttVAP27-12–PttVAP27-17) (Fig. 1a). Since transgenic trees were created in this study to specifically modify expression of the Potri.019G116400 homologue in *P. tremula *×* tremuloides*, which belongs to the smaller group, we focus here on the properties of the proteins in this group.

*Populus* VAP27-12, -13, -14, -15, -16 and -17 proteins (Fig. 1a) contain each an MSP (major sperm protein) domain (predicted by SMART-EMBL) located centrally rather than towards the *N*-terminus as observed for the other VAP27 proteins, and a coiled-coil domain (CCD, after amino-acid residue 200, Coiled Coil prediction of Prabi-Gerland site; [27]), which is common in most VAP and SNARE proteins. They have no transmembrane domain (TMD) in the *C*-terminal region (predicted by Phobius; [28]).

Expression data for the *Populus VAP27-12, -13, -14, -15, -16* and* -17* genes were retrieved from the AspWood gene expression database [10] which contains high-resolution RNA sequencing data for different stages of wood development in the aspen (*P. tremula*) stem (Fig. 1b). *PttVAP27-17* displayed low expression in the phloem/cambium region of the stem, followed by a peak in the area of xylem expansion, and decreased expression during xylem maturation and cell death (Fig. 1b). This expression pattern is consistent with a function in early xylem development.

### Overexpression of *PttVAP27-17* has potential to increase growth in greenhouse-grown trees

**Fig. 2:**
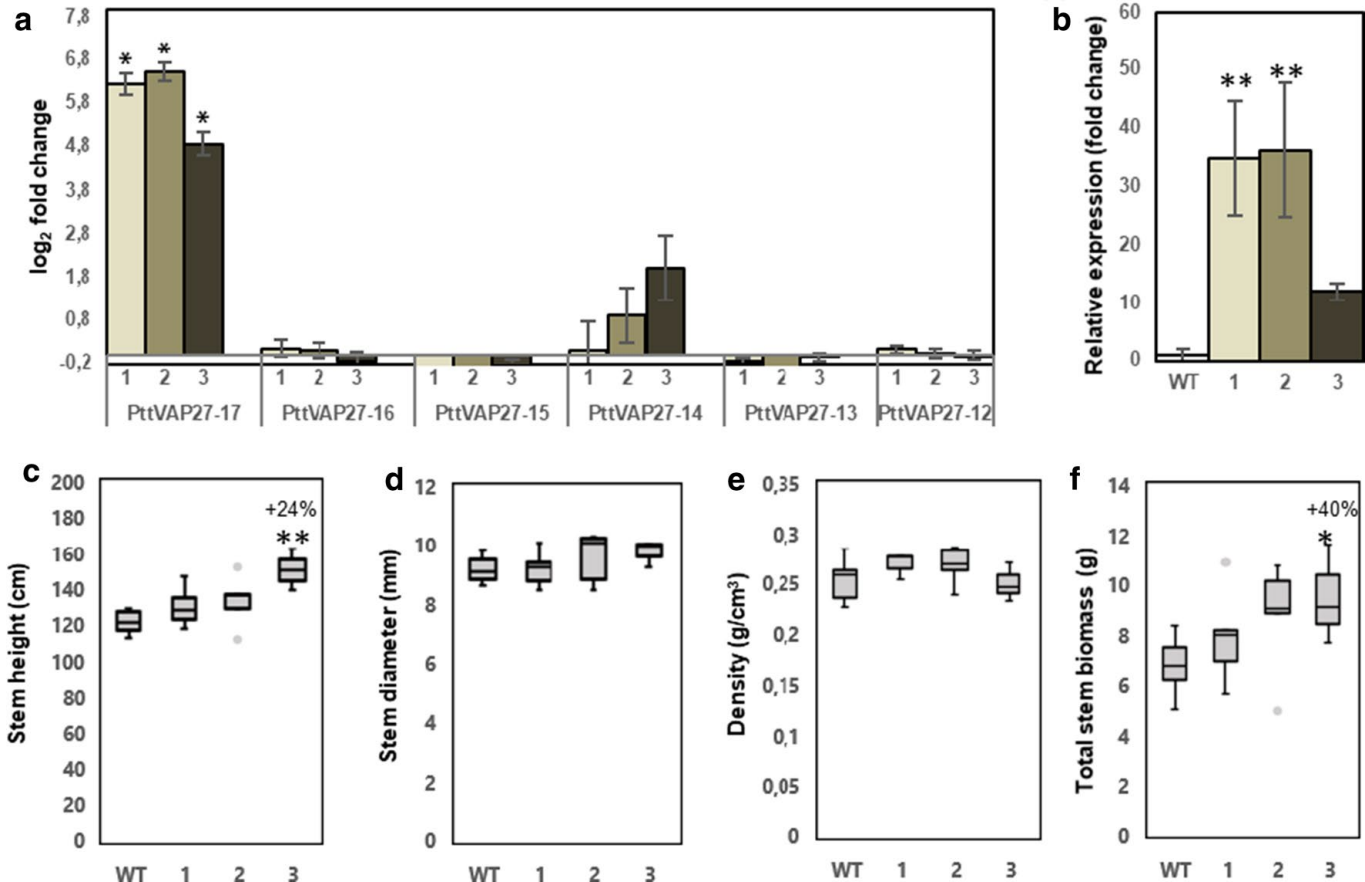
Overexpression of *PttVAP27-17* in greenhouse-grown hybrid aspen trees. **a** The expression of *PttVAP27-12, -13, -14, -15, -16* and *-17* genes in stem tissues of three transgenic *P*. *tremula* × *tremuloides* lines (1–3) carrying the *35S*::*PttVAP27-17* construct. The expression data were derived from the transcriptomic analysis of secondary xylem tissues collected from the basal part of 2-months-old greenhouse-grown trees. The log2 fold-change indicates log ratio of *PttVAP27-17* expression in each transgenic line compared to WT. The figure also shows relative expression of *PttVAP27-17* by qPCR (**b**), stem height (**c**), stem diameter (**d**), wood density (**e**), and total stem biomass (**f**) in the three transgenic *P*. *tremula* × *tremuloides* lines carrying the *35S*::*PttVAP27-17* construct and the WT grown for two months in the greenhouse. Stem volume was estimated using the formula: volume = *π* × radius^2^ × height/3. *n* = 5 for transgenic line 1 and 2, *n* = 3 for transgenic line 3 and *n* = 7 for the WT where “*n*” indicates number of biological replicates. Vertical bars (in **a**, **b**) indicate ± SD. The box plots show the median (horizontal line) with the outer limits at the 25th and 75th percentiles, the 1.5 inter-quartile ranges (whiskers) and the outliers (gray spots). Asterisks indicate statistically significant differences compared to WT: *p* ≤ 5% (*), *p* ≤ 0.1% (**), using Student’s *t*-test (**b**, **c**, **f**) and differential expression analysis DESeq2 (**a**). Percentages indicate increase (+) or decrease (−) of corresponding data values for lines overexpressing *PttVAP27-17* compared to WT

Transgenic *PttVAP27-17* overexpression lines, carrying a construct with the *PttVAP27-17* gene under the control of the constitutive CaMV *35S* promoter, were analyzed in detail for their performance in greenhouse conditions. Three transgenic *P. tremula *×* tremuloides* lines (lines 1, 2, and 3) were grown for two months in the greenhouse. The expression of *PttVAP27-17* was increased by 75-, 92- and 29-fold in the transgenic lines 1, 2, and 3, respectively, compared to the wild type (WT) (Fig. 2a). Quantitative PCR results confirmed these results (Fig. 2b). Overexpression of *PttVAP27-17* did not result in statistically significant differences in the expression of the other closely related *P. tremula *×* tremuloides* VAP27 genes (Fig. 2a).

After two months of growth in the greenhouse, all three transgenic lines showed trends towards increased growth even though statistically significant differences were found only for line 3 (Fig. 2c–f).

**Fig. 3:**
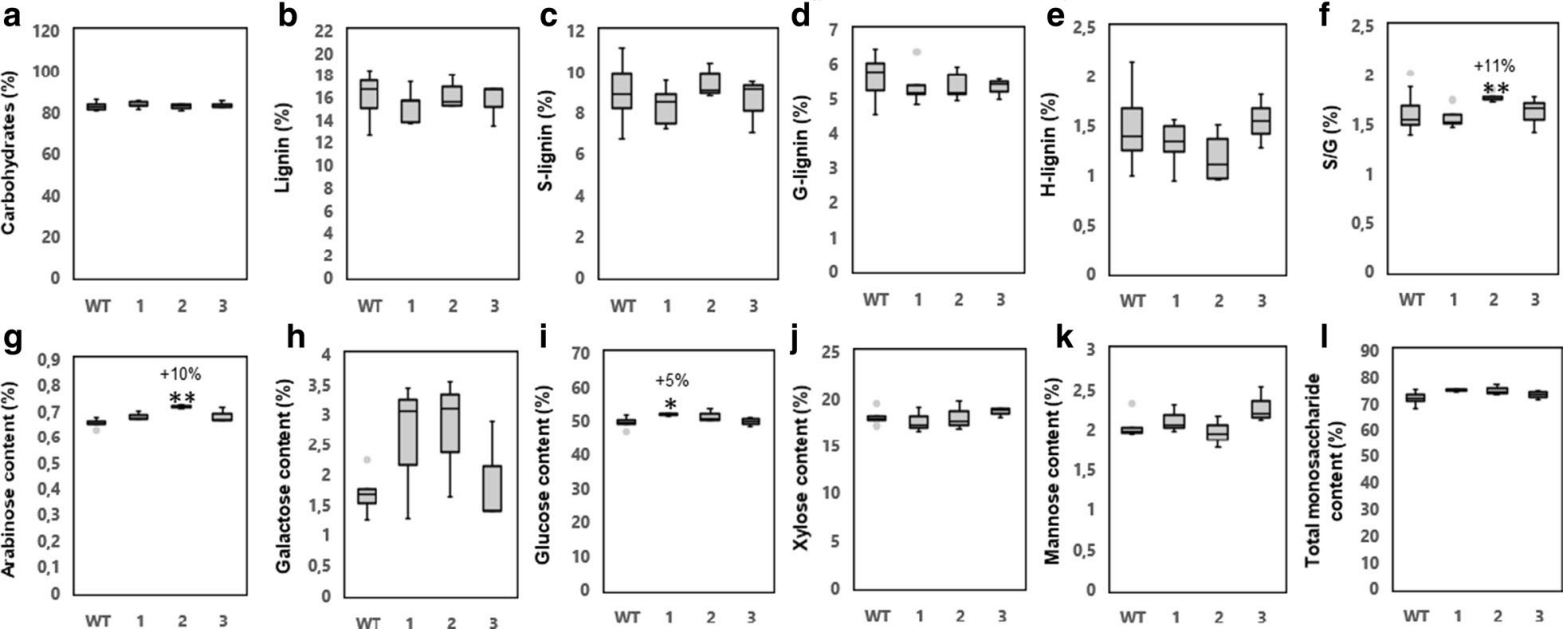
Overexpression of *PttVAP27-17* did not cause major changes in wood chemistry of greenhouse-grown trees. **a**–**f** Pyrolysis–gas chromatography/mass spectrometry analysis of cell wall components in wild type (WT) and three *PttVAP27-17* lines (1–3). The values are relative, and depict the combined area of GC peaks assigned to carbohydrates (C), lignin (L), syringyl lignin (S-lignin), guaiacyl lignin (G-lignin) and *p*-hydroxyphenyl lignin (H-lignin) as a percentage of total GC peak area. S/G, ratio between syringyl and guaiacyl-type lignin. **g**–**l** Total carbohydrate content in WT and *PttVAP27-17* lines measured by high-performance anion-exchange chromatography. The fractions of arabinose, galactose, glucose, xylose, mannose, and total monosaccharides (arabinose + galactose + glucose + xylose + mannose) after acid hydrolysis are indicated as percentages (g of monosaccharide sugar in anhydrous form per 100 g of dry weight of wood). Wood chemical analyses were performed using mature wood from the basal part of the stem of two-months-old trees grown in the greenhouse. For both type of analyses, *n* = 5 for transgenic line 1 and 2, *n* = 3 for transgenic line 3 where “*n*” indicates number of biological replicates. For the WT, 33 biological replicates were used without pooling for pyrolysis–gas chromatography/mass spectrometry analysis and with pooling to five replicates for total carbohydrate content analysis. The box plots show the median (horizontal line) with the outer limits at the 25th and 75th percentiles, the 1.5 inter-quartile ranges (whiskers) and the outliers (gray spots). Asterisks indicate significant differences from the WT at *p* ≤ 5% (*) and *p* ≤ 0.1% (**) according to Student’s *t*-test. Percentages indicate increase (+) or decrease (−) of values of lines overexpressing *PttVAP27-17* in comparison to WT

Wood chemical analyses using mature wood from the basal part of the stems were performed with two different methods, Py–GC/MS (pyrolysis–gas chromatography/mass spectrometry) and by high-performance anion-exchange chromatography after hydrolysis of carbohydrates with sulfuric acid. Py–GC/MS, which defines the abundance of wood pyrolytic products as GC peak areas in relation to the total peak area, did not reveal any significant differences even though a tendency towards increased total carbohydrate content and decreased lignin content was observed in the transgenic lines (Fig. 3a, b and Additional file 1: Table S2). Compositional analysis indicated that the transgenic lines displayed a tendency towards increased glucan content, which was statistically significant for line 1 (Fig. 3i). Altogether, these analyses demonstrate that the increased biomass production of the transgenic lines coincided with minor increases in the carbohydrate constituents and minor decreases of the lignin content of the cell walls.

### Overexpression of *PttVAP27-17* has an impact on saccharification efficiency in greenhouse-grown trees

**Fig. 4:**
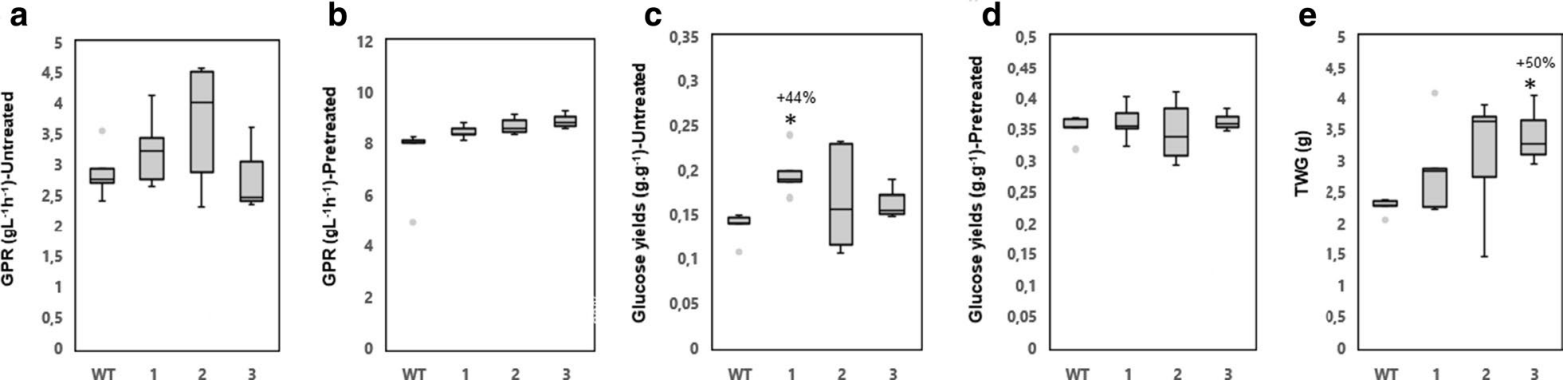
The impact of the overexpression of *PttVAP27-17* on the enzymatic saccharification of greenhouse-grown trees. **a**, **b** Glucose production rate (GPR) after 2 h of enzymatic saccharification (gL^−1^ h^−1^) for samples without (untreated) and with acid pretreatment (pretreated) in wild type (WT) and three transgenic lines (1–3). **c**, **d** Glucose yields after 72 h of enzymatic saccharification [g glucose per g of wood (dry weight) in samples without (untreated) and after acid pretreatment (pretreated)]. **e** Total wood glucose yield (TWG) after pretreatment and 72 h of enzymatic saccharification per whole tree biomass. TWG was calculated by using the formula 1/3 × *π* × stem radius^2^ × stem height × wood density × glucose yield after pretreatment and 72 h of enzymatic saccharification. *n* = 5 for transgenic line 1 and 2, *n* = 3 for transgenic line 3 and *n* = 33 for the WT where “*n*” indicates number of biological replicates. Samples from the 33 WT trees were pooled to five replicate samples in all these experiments. The box plots show the median (horizontal line) with the outer limits at the 25th and 75th percentiles, the 1.5 inter-quartile ranges (whiskers) and the outliers (gray spots). Asterisks indicate significant differences from the WT at *p* ≤ 5% (*) according to Student’s *t*-test. Percentages indicate increase (+) or decrease (−) of values for lines overexpressing *PttVAP27-17* compared to the WT

An important parameter for biotechnology purposes is susceptibility of cell wall carbohydrates to enzymatic saccharification. We, therefore, investigated whether overexpression of *PttVAP27-17* influenced the analytical-scale enzymatic saccharification with or without a prior hydrothermal pretreatment step with sulfuric acid. The principal target of this type of pretreatment is the hemicellulose. The pretreatment resulted in a liquid phase, referred to as pretreatment liquid or hemicellulosic hydrolysate. As expected, xylose was the predominant sugar in the liquid phase (Additional file 1: Table S11a). Subsequent enzymatic saccharification of the solid phase after pretreatment resulted in an enzymatic hydrolysate in which glucose was the predominant sugar (Additional file 1: Table S11a). The yields of glucose after enzymatic saccharification of pretreated solid phase were not altered in the transgenic lines compared to the WT (Fig. 4d and Additional file 1: Table S11a). However, the glucose yields after enzymatic saccharification of unpretreated wood were increased by 20–44% for the transgenic lines compared to the WT even though it was statistically significant only in line 1 (Fig. 4c and Additional file 1: Table S11a).

Another important parameter is the rate of glucose production during saccharification. We measured the glucose production rate (GPR) after 2 h of enzymatic hydrolysis with and without acid pretreatment. For all transgenic lines, the GPR increased by 13–16% compared to the WT after enzymatic hydrolysis with and without pretreatment, even though it was not statistically significant (Fig. 4a, b). Overall, the glucose yields after enzymatic hydrolysis without pretreatment suggest that the genetic modification caused by overexpression of *PttVAP27-17* affected cell wall components in a way that made the cellulose more easily accessible to enzymatic hydrolysis.

Finally, the total-wood glucose (TWG) yield was estimated. TWG estimates glucose yield after pretreatment and 72 h enzymatic hydrolysis per total stem woody biomass, and hence integrates saccharification properties (per gram dry weight) with biomass production of trees [22]. All transgenic lines had higher TWG compared to the WT, but it was statistically significant only for line 3 (Fig. 4e). The saccharification results are in agreement with our previous study, where the VAP27-17 overproduction line 3 (called “BI 36” in [22]) was identified as one of the lines with highest TWG [22]. Increased TWG in the transgenic line 3 demonstrates that saccharification yields can be significantly improved in trees having increased biomass production even though sugar yields per dry weight are not higher.

### A multi-omics approach provides a toolbox for the analysis of PttVAP27-17 function

Transgenic modifications aiming towards improved saccharification properties of the feedstock should not negatively influence important metabolic processes. Using the methodology described by Obudulu et al. [29], a multi-omics approach was used to characterize the overall effect of overexpression of *PttVAP27-17* on plant metabolic pathways. The analytical platforms included transcriptomics, metabolomics (UHPLC–MS, ultra-high-performance liquid chromatography–mass spectrometry), proteomics, GC–MS for monosaccharide sugars, and Py–GC/MS analyses of woody tissues from greenhouse-grown trees. An OnPLS analysis [30–33] was performed on data from the WT and combined data from the three lines overexpressing *PttVAP27-17*. OnPLS separates data from each analytical platform into three different parts depending on whether the data variation is globally joint (shared between all analytical platforms), locally joint (shared between some, but not all analytical platforms), or unique (specific for one analytical platform). The analysis was introgressed into an OnPLS model, which revealed that the majority of the modeled variation was globally joint, which when combined with the locally joint variation included 67% of transcriptome variation, 65% of proteome variation, 79% of UHPLC–MS variation, 86% of GC–MS variation, and 61% of Py–GC/MS variation. An overview of OnPLS integration of the five platforms using correlation plot revealed that the significantly different variables (|*p*(CORR)|≥ 0.5) between the transgenic lines and the WT were best described on the basis of the first component of the OnPLS (Additional file 1: Fig. S1) [34].

The OnPLS analysis revealed a large number of genes that were differentially expressed but only a small number of proteins and metabolites that were changed in abundance in the lines overexpressing *PttVAP27-17* compared to the WT (Table 1). Several genes annotated with microtubule-related functions were more expressed in the transgenic lines compared to the WT (Additional file 1: Tables S5–S7). Also gene ontology (GO) enrichment analysis (using the tool REVIGO from Popgenie home page [35]) of the significantly differentially expressed genes and proteins (|*p*(CORR)|≥ 0.5) revealed enrichment of genes and proteins involved in microtubule-based processes or movement of cellular components in the transgenic lines (Additional file 1: Tables S9 and S10). Other cellular components that dominated in the GO analyses were associated with non-membrane bound organelles and the (clathrin) vesicle part/coat. Also, a few other biological processes, such as (purine) ribonucleoside and ribonucleotide metabolism/binding, glycolysis, monosaccharide metabolism, and carbohydrate catabolism appeared in the GO analysis of differentially expressed genes and proteins. Taken together, the multi-omic analyses revealed both changes that one would expect as a result of a VAP27 protein overexpression as well as alterations in metabolic pathways that are not previously connected to VAP27 function. It is possible that some of these changes in metabolic pathways are secondary due to changes in the growth of the *PttVAP27-17* overexpressing trees, but the possibility that they are causally related to these changes cannot be excluded. In any case, the rather small number of metabolic pathway proteins that were significantly altered in the transgenic lines (Table 1 and Additional file 1: Table S7) suggests that *PttVAP27-17* overexpression had rather limited effects on major metabolic processes.

**Table 1:** Number of significantly different variables (|*p*(CORR)|≥ 0.5) between the *PttVAP27-17* overexpressed lines and the WT of the greenhouse-grown trees on the basis of the first globally joint component of the OnPLS analysis

Analytical platform	Total number of variables	Number of variables significantly down-regulated in transgenic lines compared to WT	Number of variables significantly up-regulated in transgenic lines compared to WT
Transcriptomics	26,444	2105	2616
Proteomics	928	2	85
Metabolites (UHPLC–MS)	992	37	101
Metabolites (Py–GC/MS)	109	2	5
Metabolites (GC–MS)	213	2	30

### Overexpression of PttVAP27-17 can increase total biomass production also in field-grown transgenic trees

Greenhouse experiments were validated in field trials in southern Sweden. The transgenic trees were grown together with WT trees for five growing seasons (August 2011–August 2016). An additional transgenic line, line 4, was added to this trial. Growth was measured twice during each growing season, and trees were analyzed with regard to wood properties at the end of the growth trial.

**Fig. 5:**
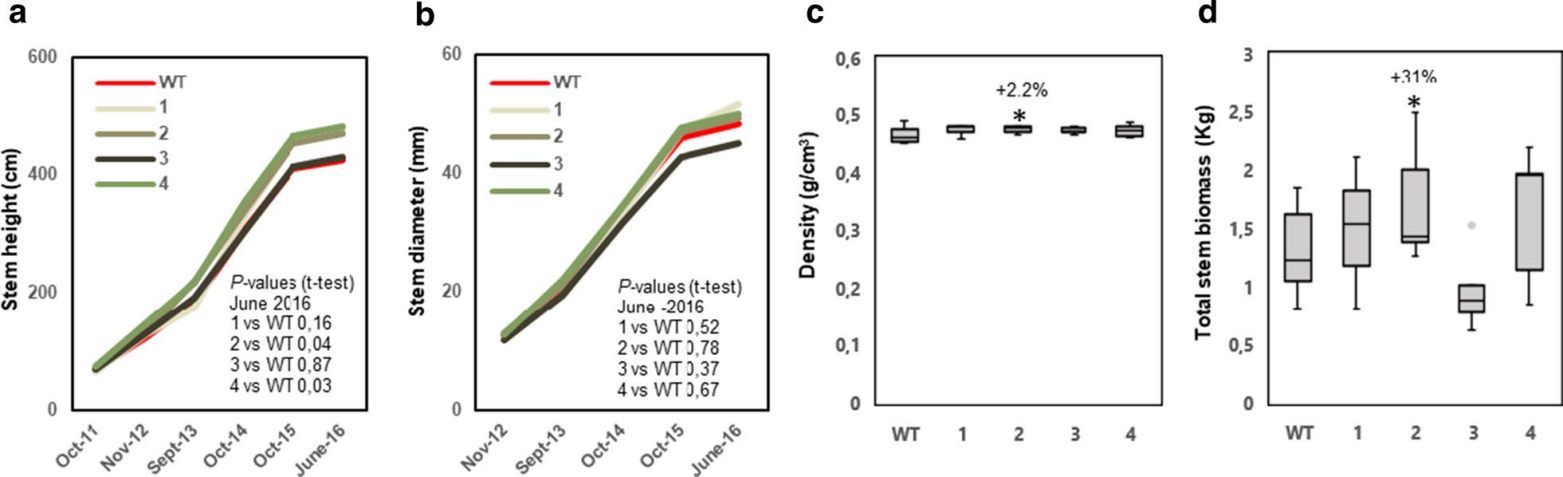
Overexpression of *PttVAP27-17* can increase stem biomass in field-grown trees. Stem height (**a**), stem diameter (**b**), wood density (**c**) at the base of the stem and total stem biomass (**d**) in field-grown trees of the wild type and four transgenic lines (1–4) during four seasons of growth (**a**, **b**) or at the end of the 5-year field trial (**c**, **d**). *n* = 3 for transgenic line 1, *n* = 5 for transgenic line 2, 3, 4 and 19 for the WT where “*n*” indicates number of biological replicates. The box plots show the median (horizontal line) with the outer limits at the 25th and 75th percentiles, the 1.5 inter-quartile ranges (whiskers) and the outliers (gray spots). Asterisks indicate significant differences compared to the WT at *p* ≤ 5% (*), according to Student’s *t*-test. The volumes of the stems were estimated using the formula: volume = *π* × radius^2^ × height/3. Total stem biomass was defined on the basis of the stem volume and density. Percentages indicate increase (+) or decrease (−) for lines overexpressing *PttVAP27-17* in comparison to the WT

Similar to the greenhouse-grown trees, overexpression of *PttVAP27-17* showed potential to increase growth of the trees in the field. Compared to the WT, lines 2 and 4 had increased stem height after the 5-year growth period (Fig. 5a). The stem diameter was not altered significantly (Fig. 5b). All lines tended to have increased wood density, which together with the increase in stem height resulted in increased total stem biomass even though it was statistically significant only for line 2 (Fig. 5c).

**Fig. 6:**
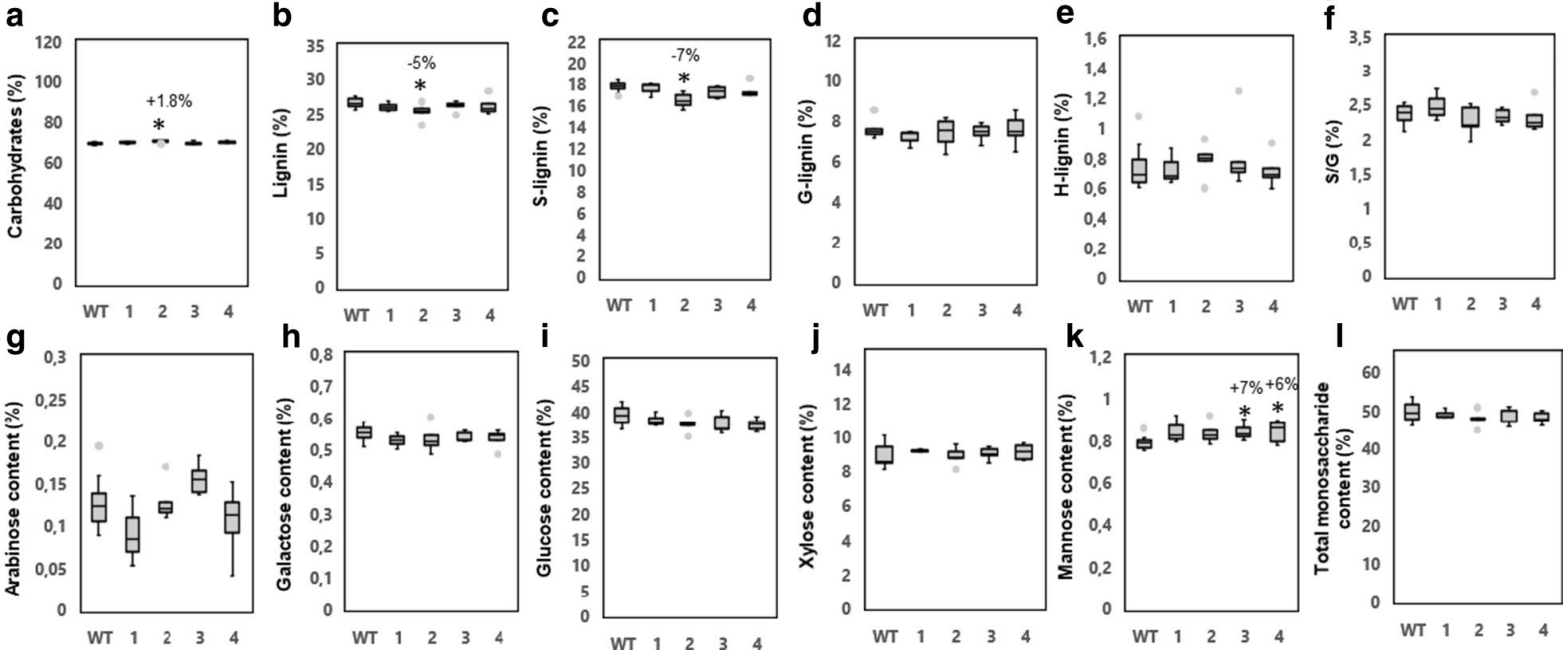
Overexpression of *PttVAP27-17* affected only slightly the wood chemistry of field-grown trees. **a**–**f** Py–GC/MS analysis of cell wall components in WT and four *PttVAP27-17* lines (1–4). The values are relative, and depict the combined area of GC peaks assigned to carbohydrates (C), lignin (L), syringyl lignin (S-lignin), guaiacyl lignin (G-lignin) and *p*-hydroxyphenyl lignin (H-lignin) as a percentage of total GC peak area. S/G, ratio between syringyl and guaiacyl-type lignin. **g**–**l** Total carbohydrate content for WT and *PttVAP27-17* lines 1–4 determined by high-performance anion-exchange chromatography. The fractions of arabinose, galactose, glucose, xylose, mannose, and total monosaccharides (arabinose + galactose + glucose + xylose + mannose) released after acid hydrolysis are indicated as percentages (g of monosaccharide sugar in anhydrous form per 100 g of dry weight of wood). For all analyses, mature wood was collected from the base of the stem of 5-year-old trees grown in the field. *n* = 3 for transgenic line 1, *n* = 5 for transgenic line 2, 3, 4 and *n* = 8 for the WT where “*n*” indicates number of biological replicates. The box plots show the median (horizontal line) with the outer limits at the 25th and 75th percentiles, the 1.5 inter-quartile ranges (whiskers) and the outliers (gray spots). Asterisks indicate significant differences from the WT at *p* ≤ 5% (*) according to Student’s *t*-test. Percentages indicate increase (+) or decrease (−) in values for lines overexpressing *PttVAP27-17* compared to the WT

**Fig. 7:**
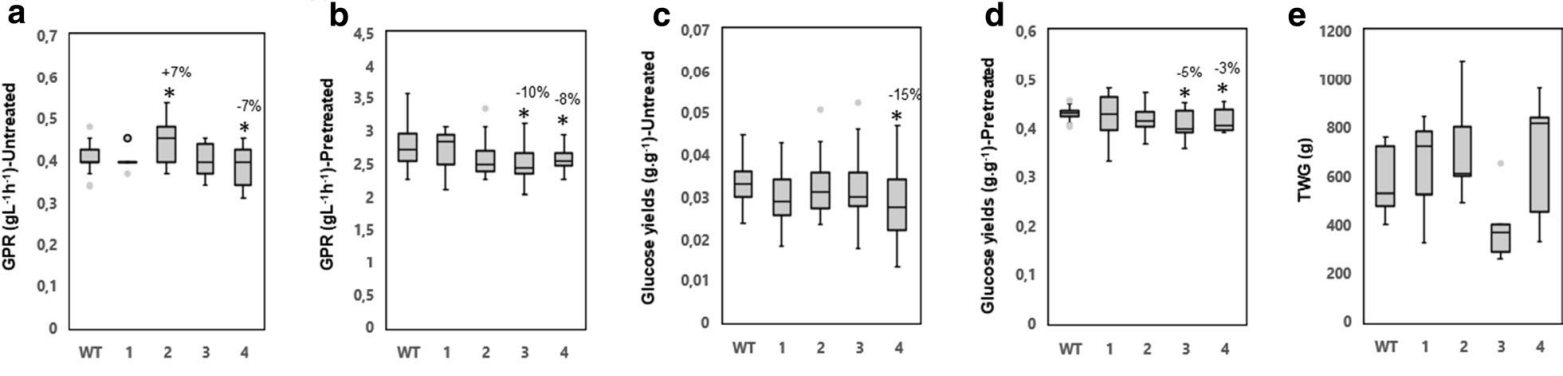
The impact of the overexpression of *PttVAP27-17* on the enzymatic saccharification in woody tissues of field-grown trees. **a**, **b** Glucose production rate (GPR) after 2 h of enzymatic saccharification (gL^−1^ h^−1^), for samples without (untreated) and with acid pretreatment (pretreated) in wild type and four transgenic lines (1–4). **c**,** d** Glucose yields after 72 h of enzymatic saccharification [g glucose per g of wood (dry weight) without (untreated) and with acid pretreatment (pretreated)]. **e** Total wood glucose yield (TWG) after pretreatment and 72 h of enzymatic saccharification per whole tree stem biomass. TWG was calculated by using the formula 1/3 × *π* × radius^2^ × height × wood density × glucose yield after pretreatment and 72 h of enzymatic saccharification. *n* = 3 for transgenic line 1, *n* = 5 for transgenic line 2, 3, 4 and *n* = 8 for the WT; where “*n*” indicates number of biological replicates. Mature wood was collected from the base of the stem of 5-year-old trees grown in the field. The box plots show the median (horizontal line) with the outer limits at the 25th and 75th percentiles, the 1.5 inter-quartile ranges (whiskers) and the outliers (gray spots). Asterisks indicate significant differences from the WT at *p* ≤ 5% (*), according to Student’s *t*-test. Percentages indicate increase (+) or decrease (−) in values for lines overexpressing *PttVAP27-17* compared to the WT

Total carbohydrate and Py–GC/MS analyses did not reveal any consistent changes in wood chemistry among the *PttVAP27-17* overexpression lines compared to the WT (Fig. 6). A tendency towards lower lignin content was present in all transgenic lines compared to the WT (Fig. 6b). Unlike the greenhouse-grown trees, the saccharification efficiency of the field-grown trees did not show any improvement, and some of the lines had even lower sugar yields compared to WT (Fig. 7a–d and Additional file 1: Table S11b). However, the total-wood glucose yield (TWG) tended to increase (by 9–24%) in three out of the four *PttVAP27-17* lines (Fig. 7e).

## Discussion

Our study shows that overexpression of *PttVAP27-17* has potential to improve growth and biomass production not only in greenhouse conditions but also in the field, even though the results varied between the transgenic lines in the two different growth conditions (Figs. 2 and 5). Several studies have demonstrated increased growth of genetically modified trees or specific tree genotypes [13, 15, 36]. However, our study is one of the few where transgenic trees have also been tested in field conditions and where data are shown for several independent transgenic lines. In other studies, trees that displayed increased growth in the greenhouse performed either the same or worse in the field [19, 37]. Our results stand out considering that the conditions in the field were quite uneven, as no extra irrigation was provided to the trees. The genotype used in these studies (T89) is also poorly adapted to the ambient conditions in southern Sweden as it originates from the Czech Republic. One of the transgenic *PttVAP27-17* overexpression lines (line 3) grew quite differently in the greenhouse and in the field. While line 3 performed best among the three lines in the greenhouse, it had the lowest biomass production among the four tested lines in the field conditions. The reason for this is not known, but it emphasizes the importance of testing transgenic lines also in field conditions to find the best-suited material for downstream processes.

Analyses of the transgenic *PttVAP27-17* trees revealed slightly increased saccharification rates and glucose yields in the greenhouse conditions (Fig. 4a–d). These trends were only observed without pretreatment, which could be related to tendencies towards lower lignin accumulation of the transgenic lines and hence increased accessibility of cellulose to enzymatic saccharification (Figs. 3b, 6b). Also, metabolic profiles suggested a decrease in lignin-specific oligomers, such as 5-*O*-4-coumaroyl shikimate, 5-*O*-caffeoyl shikimic acid and 6-hydroxy-2-cyclohexen-on-oyl sinapic acid (HCH-sinapic acid) in the transgenic trees (Additional file 1: Table S3). However, the saccharification efficiency was not improved in the transgenic trees collected from the field (Fig. 7a–d). But due to the slightly higher biomass production of the trees, the whole tree saccharification efficiency, as measured by the total wood glucose yield, tended to increase in three field-grown lines out of four when compared to the wild type (Fig. 7e).

Saccharification assays have rarely been performed in conditions resembling natural, ambient conditions. Downregulation of cinnamoyl-CoA reductase (CCR) gene in field-grown *Populus* trees resulted in improved saccharification yields (per gram dry weight) at two field sites, but for all but one transgenic line that effect was outweighed on a whole-tree basis by reduced biomass production [19]. In another study, downregulation of the 4-coumarate:coenzyme A ligase (4CL) gene using a xylem-specific promoter resulted in reduction in biomass growth, reduction in lignin content, and a negative impact on the saccharification efficiency in field-grown hybrid poplar lines [37]. However, downregulation of the same gene in *P. trichocarpa* using the *35S* promoter resulted in increased saccharification rates, but no description on tree growth was presented [38, 39]. The current study is, therefore, unique in the sense that it provides evidence from field-grown *Populus* trees on the potential of *PttVAP27-17* overexpression to increase saccharification yields without concomitant growth penalties.

Even though this study did not aim to investigate the exact function of PttVAP27-17, our multi-omic results support its involvement in microtubule organization. In line with this, an ER-associated *Arabidopsis* VAP27-1 protein was shown to bind to microtubules in vitro, suggesting that it is involved in ER anchoring to microtubules [40]. Our proteomic analyses also revealed alterations in the abundance of proteins related to vesicle trafficking and clathrin-coated vesicles, supporting a function of PttVAP27-17 in endocytosis. *Arabidopsis* VAP27-1 and VAP27-3 were recently shown to interact with clathrin and phosphatidyl inositol-phosphate (PIP) lipids [41]. It was proposed that these ER-localized VAP proteins are required for tethering ER with the PIP-enriched domains of the plasma membrane to maintain endocytic trafficking. It is possible that PttVAP27-17 is involved in a similar kind of process even though PttVAP27-17 seems to have a predominant localization in the plasma membrane, in analogy with its closest *Arabidopsis* homologs VAP27-8 and -10 [25]. Therefore, the possible role of PttVAP27-17 in microtubule function and endocytosis, and how these processes influence plant growth remain to be investigated further. Our datasets on *PttVAP27-17*-related transcriptomics, proteomics, and metabolomics provide a toolbox for these purposes.

## Conclusions

The study reports on an alternative strategy to increase saccharification yields by increasing the biomass production of trees while maintaining adequate saccharification rates. Overexpression of *PttVAP27-17* in transgenic *Populus* trees was identified as a promising tool for such a strategy on the basis of experiments performed both in the greenhouse and on field conditions.

## Methods

### Generation of greenhouse-grown transgenic plants and tissue sampling

Transgenic lines were produced by SweTree Technologies AB, Sweden, as a part of their large-scale gene-mining program in hybrid aspen (*Populus tremula* × *tremuloides*). The *PttVAP27-17* overexpression construct was created by amplifying a fragment from *Populus tremula* × *tremuloides* cDNA corresponding to gene model Potri.019G116400 with the forward primer GAAAGTTTAGTCTTTGCAAAATGCC and the reverse primer ACAAGTGCTTACAAGGAAAACAGG, followed by recombination into pDONOR201 and further into pK2GW7, under the control of the 35S promoter. The resulting vector was transformed into hybrid aspen (*P. tremula* × *tremuloides*) clone T89 according to Nilsson et al. [42]. Several transgenic lines were grown in the greenhouse, and four lines (1, 2, 3 and 4) were selected for this study on the basis of the greatest separation in growth from a large population of WT trees in a PCA analysis.

Three transgenic lines (1, 2, 3; each with 3–5 trees) and the WT (T89 with 33 biological replicates) were grown in greenhouse under controlled conditions as described in Obudulu et al. [29]. Samples were collected from the basal part of the tree; the 10–17 cm portion from the base of the stem was used for metabolomics, proteomics, and transcriptomics studies, the 33–36 cm portion for anatomical studies [fixed in a solution containing formaldehyde (5%), acetic acid (5%), and ethanol (50%)], and the 36–46 cm portion for wood chemical analysis, saccharification studies, and density measurements. Density was measured by the water-replacement method.

### Phylogenetic analysis

The evolutionary history was inferred using the WAG substitution model [43]) in the R package phangorn (v2.2.0; [44]). Bootstrap support values in the phylogram were based on 1000 bootstrap runs. Visualization was performed in R using the ggtree extension (v1.12.7; [45]) for ggplot2 (v3.0.0; [46]). Protein domain detection was done using InterProScan (v5.27-66.0; [47]) and consequently visualized using ggplot2.

### Transcriptomics, proteomics, and metabolomics

The frozen part of the stem (10–17 cm portion from the base of the stem) was peeled from the greenhouse-grown trees, and the surface of the secondary xylem consisting of living vessels and fibers (into the depth of approximately 1 mm from the surface) was scraped. The xylem scrapings were ground to fine powder in liquid nitrogen and stored at − 80 °C for transcriptome, proteome and metabolome analysis. The analyses included 3–5 biological replicates for each of the transgenic lines, and seven WT trees that were selected with a multivariate analysis to adequately represent the whole population of 33 wild-type trees that were grown at the same time with the transgenic lines in a random fashion.

#### Transcriptome analysis

Total RNA was extracted and sequenced at the Beijing Genome Institute (China) following the protocol described by Obudulu et al. [29]. The gene expression data were aligned using STAR [48] and were normalized with variance-stabilizing transformation (VST) using the R package DESeq2 [49].

#### Proteome analysis

Following the procedure described in Obudulu et al. [29], proteome analysis was performed from 20 mg of xylem sample for the selected WT and transgenic trees. Protein identification and peptide quantification was done according to Srivastava et al. [50]. Protein abundances were calculated by taking the median from the abundances of the unique peptides that matched with a protein and that showed a significance level of 0.05 corresponding to a |*p*(CORR)|≥ 0.5 in the OnPLS analysis.

#### Metabolite profiling using GC–MS and UHPLC-MS

Metabolite analyses using GC–MS and UHPLC–MS were performed following the procedures described by Jonsson et al. [51] and Obudulu et al. [29], respectively.

### qPCR analysis

Quantitative PCR analysis was performed in the same samples that were used for the transcriptome analysis by RNAseq, according to Obudulu [29], using primers ATCCAGAATGCCCTAGTCCTGCAC and AAGTCCTTCGCCAACAACTCTGG for *PttVAP27-17* and primers GGCTAATTTTGCCGATGAGA and ACGTCCATCCCTTCAACAAC for the reference cyclophilin gene (Potri.004G168800).

### Chemical and structural analysis of wood

Freeze-dried wood of transgenic and WT trees from the 36–46 cm stem portion for the greenhouse-grown trees and 5 cm bottom stem portion for the field-grown trees was cut into small chips and milled using an A11 Basic Analytical Mill (IKA, Staufen, Germany), followed by grinding in an Ultra Centrifugal mill ZM 200 (Retsch GmbH, Haan, Germany) equipped with a 0.5 mm ring sieve. The wood powder was processed further by passing it through an analytical sieve shaker AS 200 (Retsch GmbH) to collect wood powder of particle size in the range 0.1–0.5 mm. For Py–GC/MS and sugar analysis using GC–MS after acidic methanolysis, milled wood powder was further ground into a fine powder using 12 mm grinding balls at 30 Hz for 2 min in an MM400 bead mill (Retsch GmbH).

#### Pyrolysis–gas chromatography/mass spectrometry (Py–GC/MS)

Finely milled wood powder (50 µg) from each individual transgenic (3–5 per line) and WT (33 biological replicates from the greenhouse and eight biological replicates from the field) tree was loaded into an oven pyrolyser set at 450 °C (with an interface and injector at 340 °C and 320 °C, respectively) and equipped with an autosampler (PY-2020iD and AS-1020E, FrontierLabs, Japan) connected to a GC/MS (7890A/5975C; Agilent Technologies, Kista, Sweden). The pyrolysate was separated on a DB-5MS capillary column (J&W, Agilent Techologies AB) and analyzed following the method used by Gerber et al. [52].

#### Analysis of monosugars by GC–MS after acidic methanolysis

Finely milled wood powder from all transgenic (3–5 per line) and WT (33) trees was used for monosugar analysis of the greenhouse-grown trees. Extractives were removed with 80% ethanol in HEPES buffer (4 mM, pH 7.5) for 30 min at 95 °C, followed by methanol:chloroform (1:1) extraction and two washes with acetone. To remove starch, the extractive-free wood powder was treated overnight at 37 °C with α-amylase from pig pancreas (Roche 10102814001; 100 units per 100 mg of wood) in potassium phosphate buffer (0.1 M, pH 7.0). For the quantification of monosugars, 0.5 mg of amylase-treated extractive-free wood was methanolyzed using 2 M HCl/MeOH [at 85 °C for 24 h; inositol (10 μg) as internal standard] followed by trisil reagent (1,1,1,3,3,3-hexamethyldisilazane + trimethylchlorosilane + pyridine, 3:1:9) derivatization using the Sylon HTP kit (Supelco; Sigma Aldrich) [53]. The monosugars were separated on a J&W DB-5MS column (30 m length, 0.25 mm diameter, 0.25 μm film thickness) (Agilent Technologies) and determined using GC–MS (7890A/5975C; Agilent Technologies).

#### Analysis of total glucan content by high-performance anion-exchange chromatography

Dry wood powder (0.1–0.5 mm particle size, 100 mg after moisture analysis using HG63 moisture analyzer, Mettler Toledo HG63, Greifensee, Switzerland) was hydrolyzed with sulfuric acid [3 mL, 72% (w/w)] for 1 h at 30 °C. The reaction mixture was diluted to 2.5% sulfuric acid using deionized water and was autoclaved for 1 h at 120 °C [54]. After centrifugation (14,000*g* for 20 min), the supernatant was collected and analyzed with respect to monosaccharide content using the ICS-3000 (Dionex, Sunnyvale, CA, USA) for greenhouse-grown plant samples and ICS-5000 (Dionex) for field-grown plant samples, following the method described by Gandla et al. [55] and Wang et al. [56]. For the analysis of greenhouse-grown trees, three experimental replicates from the pool of 3–5 biological replicates per line and 5 replicates from the pools of 33 biological replicates of WT samples were used. For the analysis of field-grown trees, 3–5 biological replicates per line and 8 biological replicates of the WT were used.

### Pretreatment, enzymatic saccharification, and analysis of reaction mixtures

Dry and sieved wood powder (0.1–0.5 mm particle size, 50 mg after moisture analysis using the HG63 moisture analyzer) was used for enzymatic saccharification with or without hydrothermal pretreatment with sulfuric acid. For the greenhouse-grown trees, each analysis included 3–5 biological replicates for each of the transgenic lines (each replicate representing wood collected from one aspen tree) and five biological replicates for the WT (each replicate representing wood pooled from 2–3 aspen trees). For the field-grown trees, each analysis included 3–5 biological replicates per line and 8 biological replicates for the WT. Pretreatment was performed using an Initiator single-mode microwave instrument (Biotage, Uppsala, Sweden) as previously described in Gandla et al. [55]). Enzymatic saccharification of samples from greenhouse-grown trees was performed using a mixture of liquid enzyme preparations (Celluclast 1.5L and Novozyme 188) as previously described in Gandla et al. [55]. Reaction mixtures were incubated at 45 °C for 72 h. Samples for analysis of sugars were withdrawn after 2 h (GPR, glucose production rate) and after 72 h (sugar yields). Enzymatic saccharification of wood powder from field-grown trees was performed similarly, but using 5 mg of the liquid enzyme preparation Cellic CTec-2 (obtained from Sigma-Aldrich, St. Louis, MO, USA). The monosaccharide concentrations [Arabinose (Ara), Galactose (Gal), Glucose (Glu), Xylose (Xyl), and Mannose (Man)] in the pretreatment liquids and in the samples taken after 72 h were determined using an HPAEC system with pulsed amperometric detection (Ion Chromatography System ICS-3000 for greenhouse-grown plant samples, and ICS-5000 for field grown) following the procedure described by Gandla et al. [55] and Wang et al. [56]. The glucose production rate after 2 h of enzymatic saccharification was determined using an Accu-Chek^®^Aviva glucometer (Roche Diagnostics Scandinavia AB, Bromma, Sweden) as previously described by Gandla et al. [55, 57].

### OnPLS analysis

Multivariate data obtained from transcriptomics, proteomics, Py–GC/MS, UHPLC–MS, and GC–MS platforms were integrated using OnPLS following the procedures described by Obudulu et al. [29]. WT data set values were used as internal reference. Data set values from transgenic lines were combined into one dataset and normalized by subtracting the average WT value from the value of each data point of transgenics and dividing by the standard deviation (SD) of the WT. A significance level corresponding to |*p*(CORR)|≥ 0.5 was used as cut-off value to identify statistically significant variation between transgenic lines and WT [58–62]. *p*(CORR) stands for the correlation of *p* (weight of each trait within the model) with the modeled class designation between values from 0 to (±)1.

### Field trials

Field trials with the transgenic lines were conducted in a location in Våxtorp in Southern Sweden (GPS coordinates as registered at Jordbruksverket system RT90: X 1,331,589, Y 6,257,832; Våxtorp Plant nursery according to Google Maps 56° 25′ 18.6ʺ N 13° 04′ 37.9 ʺ E) during five growing seasons. The trial included eight trees for each of four different transgenic lines (1, 2, 3, and 4) and 147 WT (T89) trees. The trees were multiplied in tissue culture, and transplanted outdoors in Umeå during the summer 2011. After this period, which aimed at hardening the trees to outdoor conditions, the trees were transferred and planted in Våxtorp in the beginning of August 2011. The trees were planted randomly in eight different blocks. First measurement of the trees was conducted in October, 2011, followed by measurement of height and diameter twice a year both in the beginning and at the end of the growing season. Final measurements of height and diameter as well as material harvesting took place in August, 2016. The trees experienced severe drought stress during summer 2016, and therefore the growth measurements done in June 2016 were used as the final results of the whole trial. Damaged trees were excluded from analysis. A filter with a minimum criterion of performance were used to filter away the most damaged plants. Only trees that performed well in the filtration process were included in the analyses of tree height and diameter according to the criteria of having minimum 10 cm height increase during each growing season and a maximum of 5 cm growth reduction between consecutive measurements. After filtering, the average seasonal height increase varied between 26 and 49 cm in all trees of this trial during the four seasons of 2012–2015.

At the end of the field trial, a subset of the trees was harvested for the analyses of total biomass, wood density, wood chemistry and saccharification on the basis of having stem volume at harvest closest to the mean of the genotype. Five individuals were selected for transgenic lines 2–4, including the individual closest to the average and two both above and below the average in stem volume. For transgenic line 1, only three individuals were included since the rest did not pass the above-mentioned filtering criteria. For the wild type, 19 individuals were selected, including the individual closest to the average, and nine below as well as nine above the average in volume. Stem volume was calculated on the basis of the stem diameter and height considering the shape of the stem as a cone. For measurements of biomass production and wood density, the shoots of the current year were discarded. The fresh weight of the remaining part of the stem was measured. From the remaining stem a 5 cm piece was collected from the top of the stem and the bottom of the stem for density measurements by a water-replacement method. The dry weight of the 5 cm pieces was used to calculate total dry weight of the stem.

### Statistical analysis

Data presented in figures and tables were analyzed using the two-tailed Student’s *t*-test function in Microsoft Excel 2016 and independent sample *t*-test in IBM SPSS^®^ Statistics software using homoscedastic/equal variance to compare each individual transgenic line with the WT. Asterisks indicate significant differences from the WT at *p* ≤ 5% (*), and *p* ≤ 0.1% (**) according to Student’s *t*-test.

## Supplementary Information

**Additional file 1: Fig. S1.** Correlation matrix plot of data obtained from five platforms; GC–MS, LC–MS, Py–GC/MS, Proteomics, and Transcriptomics. **Table S1.** Monosaccharide composition of wood from wild-type (WT) and *PttVAP27-17* overexpression lines of greenhouse-grown trees. **Table S2.** Relative abundance of metabolites identified by Pyrolysis–GC/MS analysis of wild-type and transgenic *PttVAP27-17* overexpression lines grown in the greenhouse. **Table S3.** LC–MS metabolomic analysis of wild-type and *PttVAP27-17* overexpression lines of greenhouse-grown trees. **Table S4.** GC–MS metabolomic analysis of wild-type and *PttVAP27-17* overexpression lines of greenhouse-grown trees. **Table S5.** RNAseq analysis of wild-type and *PttVAP27-17* overexpression lines of greenhouse-grown trees. **Table S6.** List of all peptides identified in the proteomic study. **Table S7.** List of all proteins identified in the proteomic study. **Table S8.** Genes co-expressed with *PttVAP27-17*. **Table S9.** Gene ontology (GO) analysis of transcripts (using tool REVIGO from *Popgenie* homepage popgenie.org) with statistically significant changes in the *PttVAP27-17* overexpressing transgenic lines compared to the wild type of greenhouse-grown trees. **Table S10.** Gene ontology (GO) analysis of all proteins with statistically significant changes in the *PttVAP27-17* overexpressing transgenic lines compared to the wild type of greenhouse-grown trees. **Table S11.** (a) Overexpression of *PttVAP27-17* affects sugar yields of greenhouse-grown trees. (b) Overexpression of *PttVAP27-17* affects sugar yields of field-grown trees.

## Data Availability

The metabolomic data are deposited to the EMBL-EBI MetaboLights database (https://doi.org/10.1093/nar/gks1004. PubMed PMID: 23109552) with the identifier MTBLS628. The complete dataset can be accessed here: https://www.ebi.ac.uk/metabolights/MTBLS628. The transcriptomic data of the WT samples (accession PRJEB21452) and the transgenic samples (Accession PRJEB33910) are deposited at the European Nucleotide Archive. The mass spectrometry proteomics data have been deposited to the ProteomeXchange Consortium via the PRIDE [63] partner repository with the dataset identifier PXD023826.
